# A Method for Constructing a Loss Function for Multi-Scale Object Detection Networks

**DOI:** 10.3390/s25061738

**Published:** 2025-03-11

**Authors:** Dong Wang, Hong Zhu, Yue Zhao, Jing Shi

**Affiliations:** School of Automation and Information Engineering, Xi’an University of Technology, Xi’an 710048, China; wangdong1210@xaut.edu.cn (D.W.); zhaoyue@xaut.edu.cn (Y.Z.); shijing@xaut.edu.cn (J.S.)

**Keywords:** small-sized object detection, YOLO, predicted probability loss, feature pyramid network (FPN)

## Abstract

In object detection networks, one widely used and effective approach to address the challenge of detecting small-sized objects in images is to employ multiscale pyramid features for prediction. Based on the fundamental principles of pyramid feature extraction, shallow features with small receptive fields are responsible for predicting small-sized objects, while deep features with large receptive fields handle large-sized objects. However, during the actual network training process using this structure, the loss function only provides the error between all positive samples and labels, treating them equally without considering the relationship between the actual size of the label and the feature layer where the sample resides, which to some extent affects the object detection performance. To address this, this paper proposes a novel method for constructing a loss function, termed Predicted Probability Loss (PP-Loss). It determines the probability of each feature layer predicting the objects labeled by the labels based on the size of the labels and uses this probability to adjust the weights of different sample anchors in the loss function, thereby guiding the network training. The prediction probability values for each feature layer are obtained from a prediction probability function established on a statistical basis. The algorithm has been experimentally validated on different networks with YOLO as the core. The results show that the convergence speed and accuracy of the network during training have been improved to varying degrees.

## 1. Introduction

Object detection, as one of the core tasks in the field of computer vision, has demonstrated tremendous potential and value in various application scenarios such as autonomous driving, intelligent surveillance, and remote sensing image analysis in recent years. However, with the increasing complexity of application scenarios, the problem of small object detection has gradually become a key bottleneck restricting the performance improvement of object detection networks. In practical applications, small objects often pose challenges due to their low proportion in images and scarce feature information, making it difficult for traditional object detection algorithms to effectively capture their precise locations and category information. This, in turn, affects the overall accuracy and robustness of detection.

Especially in high-resolution images or complex background environments, the detection of small-sized objects faces severe challenges. On the one hand, small objects have a limited number of pixels, making it difficult to provide sufficient detailed features such as texture and shape for the model to learn. On the other hand, small objects are easily affected by noise and can be confused with the background, increasing the difficulty of detection. Therefore, how to design an efficient object detection network to effectively improve the detection accuracy of small-sized objects has become a hot and difficult research topic. In recent years, researchers have conducted extensive and in-depth explorations on the issue of small-sized object detection, striving to build detection models that are more sensitive and robust to small objects. They have approached this from various aspects, including improving network structures, enhancing feature representation capabilities, adopting multiscale fusion strategies, and introducing attention mechanisms.

A pyramid structure of multiscale feature maps is often mentioned as an important method. For the vast majority of feature extraction networks, as the convolutional layers deepen, the receptive field gradually expands, forming feature maps at different scales. In shallow feature maps, the receptive field is smaller, corresponding to smaller objects, while in deeper feature maps, it corresponds to larger objects. If we only analyze the shallow feature maps, it is more favorable for smaller objects. Taking ResNet as an example, the deepest P5 feature map is commonly used, and if multiscale information is required, one may also opt to include the shallower P3 and P4 feature maps.

However, deeper convolutional layers possess better feature representation capabilities. Directly using shallow features does not yield optimal results. In most cases, the FPN (Feature Pyramid Network) [1] architecture is used to fuse feature maps at different scales. FPN is a top-down approach for fusing multiscale features, and on the basis of this, subsequent researchers have proposed similar structures such as PANet [2], NAS-FPN [3], Auto-FPN [4], and BiFPN [5] among others.

In many classic object detection networks, the use of multiscale FPN (Feature Pyramid Network) for feature extraction is quite common. Representative examples include RetinaNet [6], FCOS [7], SSD [8], and the YOLO [9,10,11,12,13,14,15] series. In the YOLO series, a structure combining DarkNet [9] and FPN [1] is used for feature extraction, followed by a header structure composed of FFN (Feedforward Neural Network) to convert feature vectors into class probabilities and regressed bounding boxes. This network structure is relatively friendly to the detection of small objects. However, through statistical analysis of the validation set data, it has been found that due to the insufficient feature representation capability of shallow feature layers, the classification accuracy of such networks is significantly lower than their localization accuracy.

The main contributions of this paper are summarized as follows:

1. A new method for constructing a loss function is proposed. By establishing a prediction probability function (PPF) related to the size of the label box, and substituting the label box size into the prediction probability functions of different feature layers, the probabilities of the target object being predicted by different feature layers can be obtained. The weights of anchor points for each sample in the classification loss function are then calculated based on these probability values, thereby constructing the classification loss. In the text, this loss function is referred to as the Predictive Probability Loss (PP-Loss).
2. A statistical analysis was conducted on the prediction results of various common object detection networks with pyramid structures to determine the range of object sizes to which each feature layer is most sensitive. Based on this analysis, reasonable parameters for the prediction probability function were determined.

The remainder of this paper is organized as follows: Section 2 introduces the application of multiscale pyramid models in some mainstream networks, as well as related work on the design of loss functions for object detection. The method proposed to modify the predictive probability loss function in this paper is detailed in Section 3. Next, Section 4 introduces and analyzes the detailed experimental procedures and results. Finally, Section 5 summarizes this paper.

## 2. Related Work

### 2.1. Feature Pyramid Network in Target Detection Network

Object detection, as a cornerstone task in the field of computer vision, is of self-evident importance. Especially in the realm of small-scale object detection, traditional object detection algorithms often struggle to achieve satisfactory results due to the limited number of pixels and weak features of small objects in images. To address the challenges of small-scale object detection, a commonly adopted solution is to extract multiscale feature maps from images, utilizing a pyramid model. This model concept has been applied in many object detection networks. Some classic object detection networks that employ multiscale features include the following:

EfficientDet [5] is an efficient object detection algorithm based on EfficientNet [16]. It achieves an excellent balance between performance and efficiency by scaling network width, depth, and resolution simultaneously through a compound scaling method. For small object detection, EfficientDet employs a Feature Pyramid Network (FPN) and Bidirectional Feature Pyramid Network (BiFPN) to fuse multiscale features, enhancing the model’s ability to detect small objects. Additionally, EfficientDet introduces an Adaptive Feature Fusion (AFF) mechanism, which adaptively adjusts the feature fusion approach based on the object size, further improving the detection accuracy for small objects.

RetinaNet, with Focal Loss at its core, addresses the class imbalance problem in object detection, particularly benefiting the detection of small and hard-to-classify objects. Focal Loss reduces the loss weight of easy-to-classify samples, enabling the model to focus more on hard-to-classify samples, thereby improving the detection performance for small objects. RetinaNet also employs the Feature Pyramid Network (FPN) to fuse multiscale features, enhancing the model’s ability to perceive small objects.

CenterNet [17] is an object detection algorithm based on center point detection, which achieves object detection by predicting the location of the object’s center point, as well as its size and category. This design gives CenterNet a natural advantage in handling small objects, as the center points of small objects are easier to locate and predict. To further improve the detection accuracy of small objects, CenterNet employs techniques such as multiscale prediction and center-point heatmap regression, enhancing the model’s ability to detect small objects.

FCOS (Fully Convolutional One-Stage Object Detection) is an anchor-free object detection algorithm that achieves object detection by predicting the existence, category, and bounding box of objects on a pixel-by-pixel basis. This design avoids the complexity and hyperparameter selection issues associated with anchor boxes, making FCOS more flexible and efficient in handling small objects. To enhance the detection performance for small objects, FCOS employs a centerness branch to predict the distance from a pixel to the center of the object, thus suppressing the generation of low-quality bounding boxes and improving the detection accuracy for small objects.

The series of object detection algorithms represented by YOLO (You Only Look Once) has attracted widespread attention in both industry and academia because of its efficient and real-time detection performance. Since its introduction in 2016, the YOLO algorithm has been renowned for its end-to-end detection framework and extremely high detection speed. YOLO treats object detection as a regression problem, completing object localization and classification directly within a single neural network. This design greatly simplifies the detection process and improves the detection efficiency. To enhance the detection of multiscale objects, the YOLO network employs a feature pyramid model, accommodating objects of different sizes. On this basis, researchers have made numerous improvements to the YOLO series of object detection networks, particularly in the feature extraction module, multiscale fusion module, and prediction head module, to address the challenge of small-scale object detection. These improvements have significantly increased the detection accuracy and robustness of small objects. Furthermore, the YOLO network can be configured with different levels of complexity, exhibiting excellent adaptability. Given the advantages of the YOLO series in small object detection, this paper focuses on introducing the algorithm with an emphasis on the structure of the YOLO network.

### 2.2. The Evolution of the YOLO Network Architecture

In the YOLOv3 [11] network, significant adjustments were made to the overall structure, adopting the DarkNet [9] network as the backbone for feature extraction and introducing Spatial Pyramid Pooling [18] (SPP). In the YOLOv4 [12] network, structures such as the Path Aggregation Network [2] (PANet) were incorporated, enhancing the model’s ability to perceive small-size objects by combining features at different scales. Furthermore, CSP-Darknet53 [12] was adopted as the backbone for feature extraction, utilizing the Cross-Stage Partial Connections [19] (CSP) strategy to reduce computational load while maintaining powerful feature extraction capabilities, which is beneficial for feature representation of small-size objects. Additionally, techniques such as Mosaic [12] data augmentation and Self-Adversarial Training [12] (SAT) were introduced, improving the model’s generalization ability for small-size objects by increasing the diversity and robustness of training data. YOLOv5 [13] further optimized the YOLOv4 framework, with a particular focus on small-size object detection: it proposed an adaptive anchor box calculation scheme, where YOLOv5 automatically calculates the most suitable anchor box sizes based on the training data, aiding in more accurate matching of small-size objects and improving detection accuracy. On the other hand, to alleviate the class imbalance problem, especially when small-size object categories may be underrepresented in the dataset, YOLOv5 adopted a lightweight head design, optimizing the detection head to reduce parameters and computational load while maintaining efficient detection performance, which is particularly important for processing images containing a large number of small-size objects.

In the YOLOv8 [15] network, further improvements were made to the backbone for feature extraction. The C2f [15] module (Faster Implementation of CSP Bottleneck with 2 convolutions) was introduced into CSP-Darknet53 [19], aiming to reduce parameters by halving the number of channels. Essentially, it is a module for feature extraction that splits input data into two branches for processing. One branch is directly passed to the output, while the other branch undergoes processing through multiple Bottleneck modules. This branch design helps increase the network’s nonlinearity and representation capabilities, thereby enhancing the network’s ability to model complex data.

On the other hand, based on the YOLO network, numerous improved network algorithms have been proposed, such as ASF-YOLO [20] for feature fusion, YOLO + AFPN [21] tailored for small-sized objects, and the addition of the BiFormer attention mechanism [22] in YOLOv8, among a series of other enhancements.

### 2.3. The Evolution of Loss Functions

With regard to the design of the loss function in the YOLO series of networks, it has undergone a series of evolutionary changes. In the networks following YOLOv5, a free-anchor structure is basically adopted, which does not constrain the shape of the target box corresponding to the anchor. The prediction outputs of anchors that fall within the labeled box are treated as positive samples to calculate the loss function. The loss function is defined as follows:

(1) $$Loss=\lambda_{1}\times L_{reg}+\lambda_{2}\times L_{class}$$

The total loss is a weighted sum of the classification loss and the regression loss, with specific weights that may be adjusted depending on the task and datasets. Here, $\lambda_{1}$ and $\lambda_{2}$ are the weight coefficients for the classification loss and the regression loss, respectively. Typically, the regression loss employs IOU loss, while the classification loss uses binary cross-entropy loss.

For the regression loss function, the IoU (Intersection over Union) loss function measures the degree of overlap by calculating the ratio of the intersection to the union of the predicted box and the ground truth box. Subsequent improvements to the IoU loss function led to the proposal of a series of loss functions such as GIoU [23], DIoU [24], and CIoU [24]. Among these, the CIoU (Complete Interaction over Union) loss function is commonly used in versions after YOLOv5. CIoU not only considers the degree of overlap but also takes into account the size and position information of the bounding boxes, the distance between their center points, and the difference in aspect ratio, which can better guide the network to learn accurate bounding boxes. Based on this, subsequent researchers have further proposed regression loss functions for bounding boxes such as MPDIoU [25], Powerful-IoU [26], Inner-IoU [27], and Scale-Sensitive IoU [28].

MPDIoU is a novel bounding box similarity metric based on the minimum point distance, which directly minimizes the distance between the top-left and bottom-right corners of the predicted bounding box and the ground truth bounding box. MPDIoU incorporates all relevant factors considered in existing loss functions, namely overlapping or nonoverlapping areas, center point distance, and aspect ratio deviation, while simplifying the computation process. MPDIoU addresses this issue by introducing Partial Distance, which refers to the distance between two boxes and can be used to measure the dissimilarity between them.

Powerful-IoU addresses the shortcomings of traditional IoU by introducing additional penalty terms or adjusting calculation methods. It can calculate the distance between the centers or boundaries of predicted boxes and ground-truth boxes, providing gradient information even when there is no overlap, thereby improving the stability of model training. Additionally, Powerful-IoU may reduce the impact of scale variations on detection results through normalization or other mathematical techniques, making it more robust in detecting objects of different scales, especially small targets.

The proposal of Inner-IoU aims to address the limitations of existing IoU loss functions, which exhibit weak generalization ability and slow convergence speed in different detection tasks. The authors propose using auxiliary bounding boxes to calculate the loss in order to accelerate the bounding-box regression process. In Inner-IoU, the authors introduce a scale factor ratio to control the size of the auxiliary bounding boxes. By using auxiliary bounding boxes of different scales for different datasets and detectors, the limitations of existing methods in terms of generalization ability can be overcome.

Scale-Sensitive IoU is a novel loss function proposed to enhance object detection, especially for multiscale targets in remote sensing images. It addresses the limitations of CIOU loss, like slow convergence and the inability to distinguish special bounding boxes. By introducing a new geometric factor (area difference) and an area regulatory factor, it adjusts loss values and boosts detection accuracy.

On the other hand, the YOLO series networks employ the binary cross-entropy loss function for the classification loss.

(2) $$L_{class}=-\sum_{i=1}^{n}\left[y_{i}log\left({\hat{y}}_{i}\right)+\left(1-y_{i}\right)log\left(1-{\hat{y}}_{i}\right)\right]$$

In the classification loss of mainstream object detection networks nowadays, this loss function is used in the vast majority of cases, indicating that its design has significant advantages in classification problems. Other loss functions, such as the mean squared error loss function, are increasingly less used in classification problems.

In other networks that adopt the pyramid model, some researchers have also improved the classification loss. For example, SBL-RetinaNet [29] uses a pre-trained network to extract features, which are then used to estimate the salience complexity of images and incorporated into the loss function. This approach improves the performance while keeping the original network structure unchanged to maintain the inference speed.

The Architecture-Agnostic Balanced Loss (ARUBA) [30] algorithm is a balanced loss function based on object size. It assigns higher weights to small-sized objects and effectively addresses the issue of object size imbalance in the datasets of UAV aerial images.

From the methods mentioned above, we can observe that most improvements to loss functions have focused on regression loss for bounding boxes. However, we find that the error rate for object classification is often higher than that for object localization. Especially in the case of small object detection, the lack of sufficient feature representations leads to a higher error rate in classification problems. In this paper, we focus on improving the classification loss function for the network, and we will introduce the specific process of each step of the algorithm in detail in the next section.

## 3. Methods

The core approach is to calculate the weight coefficients of each sample in different feature layers when computing the loss of classification during network training, based on the size of the labels. These weight coefficients are obtained by substituting the label sizes into the prediction probability functions of different feature layers. The specific implementation process is as follows.

### 3.1. The Design of the Loss Function

Taking the YOLOv8 network as an example, the loss function used consists of two parts: classification loss and regression loss. Various methods have already been used in regression loss to reflect the influence of the size of the bounding box on the loss value. This paper will focus on classification loss.

Now, we define the number of classification categories in the detection network as *N*. One of the anchor points, denoted as $A_{i}$, is selected as a positive sample, with its classification prediction output $pd\_s_{i}$ being an N-dimensional vector. This anchor point is known to be contained within a target bounding box $B_{k}$. Based on the labeled category of this target bounding box $B_{k}$, an N-dimensional binary vector can be constructed, denoted as $gt\_b_{k}$. Then we can use the binary cross-entropy loss function to calculate the classification loss for this positive sample.

(3) $$L_{i}=BCELoss\left(pd\_s_{i},gt\_b_{k}\right)$$

Ultimately, we take the average of the classification losses of multiple positive sample anchors as the overall classification loss for the network.

(4) $$L_{class}=\frac{1}{N_{pos}}\sum_{i=1}^{N_{pos}}L_{i}$$

**Figure 1:**
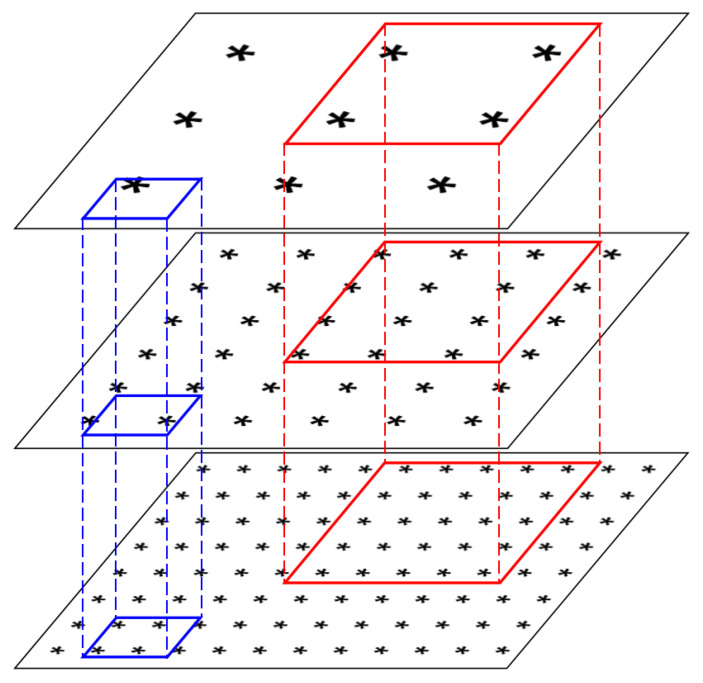
Relationship between Anchors and Target Bounding Boxes. The asterisks (*) in the figure represent preset anchors, with their size indicating which layer of the pyramid model they belong to; larger sizes represent higher-level features, and smaller sizes represent lower-level features. Each labeled target bounding box will contain multiple anchors located in different layers.

During this process, we know the actual size of the bounding box $B_{k}$, denoted as $s_{k}$, and also know which layer of the output pyramid features the anchor $A_{i}$ resides in. From Figure 1, it can be seen that regardless of the size of the target bounding box, it is possible for it to contain multiple anchor points across different feature layers. The original design intent of the Feature Pyramid Network is to use the features corresponding to anchors in deeper layers for predicting large objects (as shown in the red boxes), and the features corresponding to anchors in shallower layers for predicting small objects (as shown in the blue boxes). However, among the anchors actually contained within a certain target bounding box, we can observe that small-sized target bounding boxes may contain anchors from higher-level feature layers, and large-sized target bounding boxes may contain anchors from lower-level feature layers. Therefore, we cannot treat these anchors equally when calculating the classification loss. We hope to apply different weights to anchors located in different feature layers.

Here, we define a Prediction Probability Function (PPF) based on the size of the target bounding box (in practice, we choose the larger width and height as the target bounding box size) and the feature layer where the anchor is located. This function is used to calculate the loss weights for each sample anchor. Ultimately, we define the formula for calculating the classification loss as follows.

(5) $$L_{class}=\frac{1}{N_{pos}}\sum_{i=1}^{N_{pos}}W\left(s_{ki},l_{i}\right)BCELoss\left(pd\_s_{i},gt\_b_{k}\right)$$

Here, $N_{pos}$ represents the number of positive samples. $s_{ki}$ represents the actual dimensions of the target bounding box $B_{k}$ that contains the *i*th positive sample, $l_{i}$ indicates the level in the feature pyramid where the anchor point corresponding to the *i*th positive sample is located, and the function *W* is a function of these two, used to adjust the weights of the loss value for the anchor point.

### 3.2. The Definition of the Predictive Probability Function

Next, we focus on introducing the detailed definition of the prediction probability function *W*. Theoretically, if a target bounding box contains anchors from multiple feature layers, determining which layer’s anchors are better suited to predict it depends on the target’s size. The larger the size, the more likely it is to be predicted by deeper feature layers, whereas smaller sizes are more likely to be predicted by shallower feature layers. When multiple prediction feature layers of different sizes exist (typically ranging from 3 to 5 layers), each layer corresponds to a range of target sizes that it is most adept at predicting. Whether the target size is too large or too small, the probability of that target being predicted by the current feature layer will decrease to some extent.

From the algorithmic foundation of convolutional neural networks (CNNs) in image processing, the spatial coverage of features extractable by a specific layer is governed by many factors, including the receptive field of the feature layer, upsampled deep features, and shallow-layer feature fusion. Additionally, the introduction of appropriate contextual information is also beneficial for the detection performance of target objects.

Based on the above theory, we define a normal distribution function for each feature layer to obtain the probability that the current feature layer predicts a target object of a certain size. The number of normal distribution functions required in the final network depends on the number of output layers in the pyramid model of the network. Here, we take a pyramid model with three output feature layers as an example.

**Figure 2:**
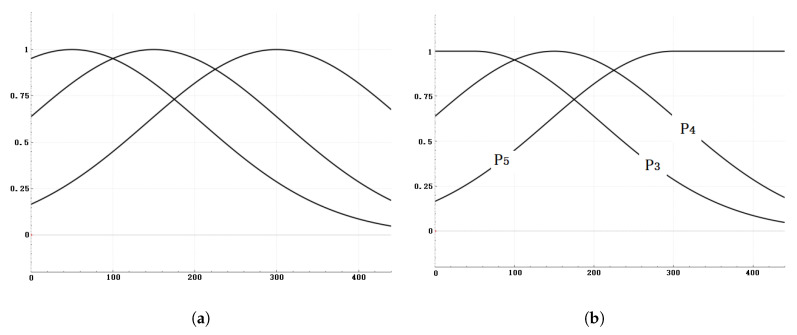
Curves of prediction probability functions for multiple feature layers. the horizontal axis corresponds to the size of the target bounding box, and the vertical axis represents the probability of being predicted by that feature layer. (**a**) The curves corresponding to the deepest layer and the shallowest layer have not been adjusted. (**b**) The curves corresponding to the deepest layer and the shallowest layer have been adjusted.

The probability prediction function is defined as follows:

(6) $$P\left(s,l\right)=e^{-\frac{{\left(s-\mu_{l}\right)}^{2}}{2{\sigma_{l}}^{2}}}$$

where *s* represents the size of the target bounding box, and *l* indicates which layer of the feature pyramid, l∈{3,4,5}. Then, we need to determine three sets of means $\mu_{l}$ and standard deviations $\sigma_{l}$. After we determine the size *s* of the target bounding box, we can substitute it into the three prediction probability functions to obtain P(s,3), P(s,4) and P(s,5). We plot the curves of the three sets of prediction probability functions on the same graph, as shown in Figure 2a. In Equation (7), special regulations are made, respectively, for the prediction probability functions corresponding to the two layers at the shallowest and deepest of the pyramid structure. Since larger targets will definitely be predicted in the feature layer at the deepest, and smaller targets will definitely be predicted in the feature layer at the shallowest, the probability should not decay. Therefore, in Equation (7), special regulations are specifically made for the prediction probability functions of the two feature layers when l=3 and l=5. The curves of the three adjusted probability functions are shown in Figure 2b.

(7) $$P\left(s,3\right)=\left\{\begin{array}{cc} e^{-\frac{{\left(s-\mu_{3}\right)}^{2}}{2{\sigma_{3}}^{2}}} & \;s\geq \mu_{3}\\ 1 & \;s<\mu_{3} \end{array}\right.\;\;\;\;\;\;\;\;\;\;\;\;P\left(s,5\right)=\left\{\begin{array}{cc} 1 & \;s\geq \mu_{5}\\ e^{-\frac{{\left(s-\mu_{5}\right)}^{2}}{2{\sigma_{5}}^{2}}} & \;s<\mu_{5} \end{array}\right.$$

Next, given a ground truth bounding box, its size *s* is used to determine the probabilities of the object being predicted by each of the three different feature layers. If we directly use these three probability values as weights to adjust the final loss function, it would shrink the original loss function because the three probability values we obtain are necessarily less than one. This is not the desired outcome. Our goal is to guide the training process toward a reasonable convergence direction while ensuring that the magnitude of the loss remains stable, without significant fluctuations compared with the original loss.

Therefore, we first substitute s into Equations (6) and (7) to obtain the probability values P(s,3), P(s,4), and P(s,5) of the object of this size being predicted by the corresponding feature layers.

Therefore, based on the obtained probability values P(s,3), P(s,4), and P(s,5), we made adjustments to obtain three weight coefficients W(s,3), W(s,4), and W(s,5). The specific formulas are as follows:

(8) $$W\left(s,l\right)=\frac{N\times P(s,l)}{\sum_{i=3}^{5}P\left(s,i\right)}\;\;\;\;\;\;\;\;\;\;\;\;\;l=3,4,5$$

Here, *l* represents which layer of the feature pyramid the selected positive sample comes from. *N* represents the total number of layers in the FPN. It can be observed that before introducing the weight coefficients, the weight coefficient for summing the losses of each positive sample point is one. Therefore, the sum of *N* weight coefficients is *N*. After applying the adjustment mentioned above, the sum of the weight coefficients remains unchanged. This essentially ensures that the magnitude of the loss function does not undergo significant changes, but rather tends to amplify the loss value of the output layer corresponding to the target box size, thereby accelerating network convergence and improving detection performance.

### 3.3. The Determination of Parameters

From the content discussed in the previous section, it can be understood that the output weights of the prediction probability functions are influenced by each set of means and standard deviations, particularly the means. So, how should we find the most reasonable parameters for the current network? For variant networks based on the YOLO architecture, different backbones and FPN structures can affect the size range of objects that each feature layer can predict. In common feature extraction networks, determining which convolutional layer can extract target object features more comprehensively while minimizing interference primarily hinges on a critical factor: the receptive field size of the corresponding convolutional layer. On the other hand, in the FPN process, deep features are upsampled and fused with shallow features, which essentially also expands the receptive field of the shallow layers to some extent. Moreover, due to the irregular and variable shapes of target objects, it is challenging to precisely determine which specific feature layer will predict a target of a given size.

To address this issue, we consider adopting a statistical approach based on actual samples to obtain the final reasonable parameters. Here, we use the YOLOv8s network as an example to briefly explain the statistical method. The statistical process for other versions of the network is similar.

We use the trained YOLOv8s network to perform object detection on all images in the datasets, obtaining each predicted object in image, which we refer to as $B_{i}\left(s_{i},l_{i}\right)$. Where *s* represents the size of the object bounding box (which is the larger of its width and height). *l* represents which feature layer the object is predicted from, l∈{3,4,5}. We separately analyze the distribution of the sizes s of the objects predicted by different feature layers. Considering that the size of the object bounding box output by the network is a noninteger, when performing the statistics, we divide every 10 pixels into a range. The predicted size falls into a certain range, and the corresponding counter is incremented accordingly.

**Figure 3:**
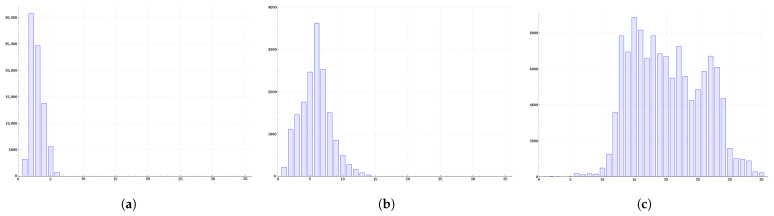
Statistical Chart of Predicted Target Sizes at Feature Layers. Subfigures (**a**–**c**), respectively, display the statistical histograms of prediction results from the $P_{3}$, $P_{4}$, and $P_{5}$ feature layers. The horizontal axis corresponds to the size of the predicted target objects, and the vertical axis represents the number of target objects predicted at that size.

To ensure that our statistical sample includes targets of various sizes as much as possible, we have incorporated both the DIOR datasets and the VisDrone datasets into our statistical scope. The results of our analysis are shown in Figure 3.

From the statistical results, it can be seen that there is a clear progressive process in the size distribution of targets predicted by different feature layers during the actual network prediction process. For the YOLOv8s network, based on the statistical results, we ultimately determined the parameters $\mu_{l}$ for the prediction probability function curves corresponding to the three layers. The respective values are $\mu_{3}=20$, $\mu_{4}=60$, $\mu_{5}=120$.

The mean value $\mu_{3}=20$ is obtained from Figure 3a. The mean value $\mu_{4}=60$ is obtained from Figure 3b. The value of $\mu_{5}=120$ is obtained from Figure 3c. As can be seen from Figure 3c, the mean value of the statistical data is approximately over 200. However, considering that $P_{5}$ corresponds to the highest layer and its final form should refer to the curve shape in Figure 2b, $\mu_{5}$ is therefore set to 120.

For other improved network architectures, there may be cases where the feature extraction layers consist of four or five layers. Similarly, a reasonable set of parameters for each feature layer can be determined by applying statistical methods to the prediction results of a pre-trained network.

For the other parameter, the standard deviation σ, we assign the same value to all layers in the network. The specific value chosen is compared and analyzed through ablation experiments in the next section.

## 4. Results

In this experiment, we employed the relatively compact YOLOv8s as the base network, with input images resized to 640 pixels. We incorporated the designed PP-Loss module into various enhanced networks, including YOLOv8s, YOLOv8s + AFPN [15,21], ASF-YOLO [20]. Meanwhile, we also conducted experiments in the four header structures of YOLOv8s. Additionally, we conducted tests on the YOLOv10s [31] network of a similar scale. The primary focus was on comparing the network performance before and after the integration of the PP-Loss module.

### 4.1. Comparative Experiment

Datasets: We performed experiments on the VisDrone [32] datasets and the DIOR [33] datasets. The models were trained on the training sets and evaluated on the validation sets. Considering the excessively high resolution of the DIOR datasets images, we split the images into subimages with a resolution of 1024 × 1024, using a 200-pixel overlapping region, for training and validation.

We compared the experimental effects of introducing or not introducing the PP-Loss component on the network, ensuring that relevant parameters such as the optimizer, base learning rate, and batch size remained unchanged throughout the process. The network was trained on an NVIDIA GTX 3090 GPU, and ultimately, we compared the results after 150 epochs of training.

From the experimental results, the introduction of PP-Loss has led to varying degrees of improvement in the object detection performance of networks with different structures, with a maximum increase in mAP of around 2%. Especially for small-sized objects, the maximum increase in the mAP is approximately 3.5% (see Table 1 and Table 2).

Our method focuses on the improvement of the classification loss function. Similar algorithms include Salience Biased Loss and Architecture-Agnostic Balanced Loss (ARUBA). We conducted a comparative experiment between the detection results of these algorithms on the validation set of the VisDrone dataset and our proposed PP-Loss (see Table 3).

**Figure 4:**
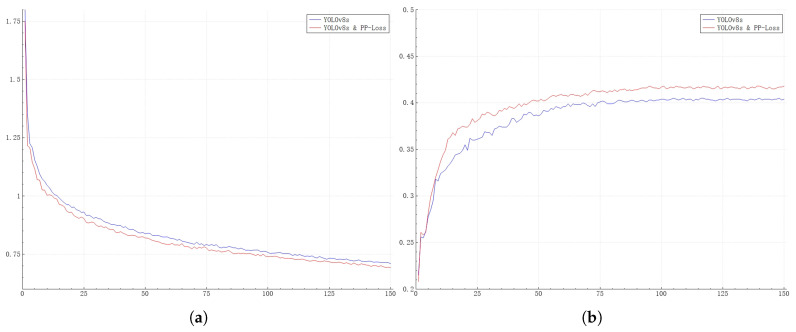
Comparison of the training processes of the YOLOv8s network before and after the introduction of PP-Loss. The data curves for 150 training epochs are plotted. (**a**) shows the classification loss curve, and (**b**) shows the mAP curve. The horizontal axis represents the epoch of the training, the vertical axis of subfigure (**a**) represents the classification loss value, and the vertical axis of subfigure (**b**) represents the mAP value.

In Figure 4, we present a comparison of the training processes of the YOLOv8s network before and after the introduction of PP-Loss. As can be seen from the curves in the figure, after the introduction of PP-Loss, not only has the performance of the network been enhanced, but also the convergence speed during network training has been improved.

For different feature layers of the FPN, after being trained by PP-Loss, they tend to predict target objects of corresponding sizes. In particular, the shallow feature layers tend to predict small target objects. To further prove this point, we statistically analyzed the prediction results of the trained network. The experimental scheme refers to the statistical method in Section 3.3. The sizes of the actual target objects predicted by the three output layers of the FPN were statistically analyzed, and the dataset used is consistent with that in Section 3.3. We display the two sets of bar charts of the same layer in the same figure.

**Figure 5:**
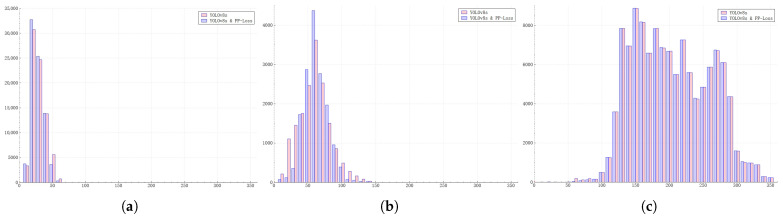
Statistical results of the sizes of target objects predicted by each feature layer of the FPN under the action of different losses. Subfigures (**a**–**c**), respectively, display the statistical histograms of prediction results from the $P_{3}$, $P_{4}$, and $P_{5}$ feature layers. The definition of the coordinate system is consistent with that of Figure 3.

As shown in Figure 5, from the distribution of the bar charts, it can be seen that the sizes of the target objects predicted by each layer in the FPN are more concentrated within a certain size range compared with the situation before the introduction of PP-Loss. In other words, the variance of the distribution of the predicted target sizes output by a single layer is smaller. This phenomenon is more obvious in the shallow-layer outputs of the FPN. On the contrary, in the deep-layer outputs that predict larger target objects, this phenomenon is not so obvious. This also shows from another perspective that the method we proposed has a more significant improvement effect on small-sized target objects.

### 4.2. The Influence of Standard Deviation σ

We conducted comparative experiments to investigate the impact of the parameter σ in the predictive probability function on the detection performance.

We trained and validated the standard YOLOv8s network on the VisDrone datasets and performed comparative experiments with the standard deviation σ in the predictive probability function set to 50, 100, 200, and 300 (see Table 4).

Through experimental comparison, we found that setting the standard deviation to 200 yields the best overall network performance. Conversely, if the value is set too small, it can adversely affect the network’s effectiveness.

**Figure 6:**
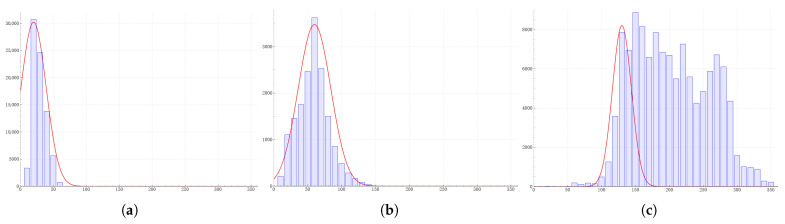
Standard deviation of the predicted probability function obtained by fitting statistical data.Subfigures (**a**–**c**), respectively, show the fitting curves when l=3, l=4, and l=5 in the FPN. Finally, based on the fitting results, the standard deviations are taken as $\sigma_{3}=19$, $\sigma_{4}=24$, and $\sigma_{5}=14$.

Next, we further optimize the selection of the standard deviation of the parameters in Equation (6). We need to explore whether it is possible to use different standard deviations for the prediction probability functions of each layer. In Section 3.3, we performed statistics on the sizes of the target objects predicted by the feature maps of each layer of the FPN and determined the mean of the probability function based on the statistical results. Similarly, we can further determine the standard deviation σ according to the statistical data. Here, we fitted the prediction probability curves for each layer, respectively, according to the statistical data and determined that the standard deviations of each prediction probability function are $\sigma_{3}=19$, $\sigma_{4}=24$, and $\sigma_{5}=14$. The curves are shown in Figure 6.

We trained the network using the parameters obtained from the fitted data, and the results are shown in Table 5. It can be seen from the comparative experiment that when the network is trained with the parameters fitted from the data, the performance drops significantly. The value of the fitted standard deviation is too small. Within most of the range of the prediction probability function, the probability values are close to zero. Not only can it not correct the original loss, but it will instead cause a certain degree of interference to it. On the other hand, the statistical results are influenced not only by the network itself but also by the dataset. And the original intention of our design is not to expect the prediction probability function to completely approximate the statistical data. Therefore, in the end, we still choose to use the same standard deviation for the prediction probability function of each layer.

### 4.3. Comparison of the Detection Results

**Figure 7:**
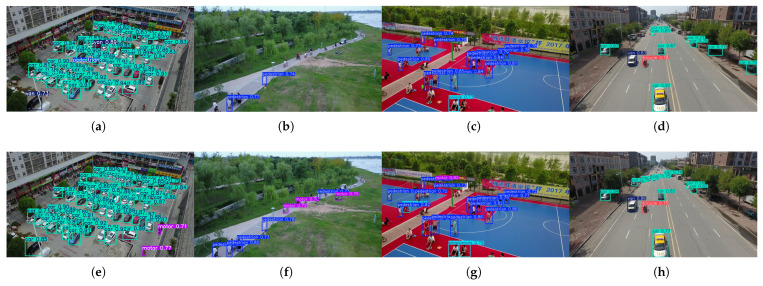
Comparison of the detection effects of the images in the VisDrone dataset. Subfigures (**a**–**d**) show the detection effects of YOLOv8s without the introduction of PP-Loss, while subfigures (**e**–**h**) demonstrate the detection effects of YOLOv8s with the introduction of PP-Loss.

Finally, we present the detection performance of the original YOLOv8s network and that of the YOLOv8s network after the introduction of PP-Loss (see Figure 7).

## 5. Discussion

In our experiments, we utilized two datasets: VisDrone and DIOR. The VisDrone dataset consists of aerial images captured by drones, containing a large number of small-size objects. The DIOR dataset is a satellite remote sensing dataset, where we split the original images into patches, resulting in a wide size distribution of ground truth boxes. As shown in Table 1 and Table 2, the detection performance of YOLOv8s and its improved variants varies slightly across these datasets. However, after introducing the PP-Loss component, training effectiveness is consistently improved, regardless of whether the pyramid structure of the network has 3 or 4 layers.

On the other hand, as shown in the experimental data in Table 3, the selection of the standard deviation σ is also crucial. If an inappropriate value is chosen, it may degrade the performance of the network, resulting in outcomes worse than those without PP-Loss. Future research could further explore selecting more reasonable values for σ or even assigning different values to different layers.

## 6. Conclusions

This paper proposes a novel method for constructing a loss function. It establishes a prediction probability function for each feature layer with respect to the label size, which calculates the probability of a target object being predicted by a certain feature layer based on its label size. This probability value is then used to determine the weight coefficients of the selected sample anchors in the computation of the classification loss. The ultimate goal is to steer the network’s convergence towards having the feature layer most suited to the object’s size predict the object. The paper provides the definition of the predictive probability function and the method for determining the relevant parameters.

The experimental results demonstrate that for a series of variant networks based on the YOLO architecture and equipped with an FPN structure, the introduction of the PP-Loss designed in this paper leads to varying degrees of improvement in the final mAP (mean Average Precision). On the other hand, by adjusting the details of this scheme, it can also be applied to other object detection networks featuring pyramid structures, such as FCOS, RetinaNet, and SSD.

## Figures and Tables

**Table 1:** Comparison of Experimental Results of VisDrone Datasets.

Method	PP-Loss	Image Size	FPN Layers	*P*	*R*	${\mathrm{mAP}}_{S}$	${\mathrm{mAP}}_{M}$	${\mathrm{mAP}}_{L}$	mAP	mAP50
YOLOv8s	No	640	3	0.520	0.392	0.103	0.513	0.719	0.241	0.404
YOLOv8s	Yes	640	3	0.532	0.399	0.126	0.518	0.719	0.258	0.417
YOLOv8s + 4H	No	640	4	0.531	0.413	0.120	0.524	0.718	0.256	0.427
YOLOv8s + 4H	Yes	640	4	0.550	0.422	0.138	0.523	0.719	0.268	0.442
YOLOv8s + AFPN	No	640	3	0.575	0.456	0.172	0.549	0.721	0.299	0.482
YOLOv8s + AFPN	Yes	640	3	0.595	0.480	0.187	0.553	0.720	0.310	0.504
ASF-YOLO	No	640	3	0.522	0.400	0.102	0.507	0.717	0.239	0.407
ASF-YOLO	Yes	640	3	0.528	0.411	0.105	0.508	0.718	0.241	0.418
YOLOv10s	No	640	3	0.512	0.382	0.101	0.509	0.721	0.237	0.393
YOLOv10s	Yes	640	3	0.534	0.407	0.124	0.514	0.724	0.256	0.419

**Table 2:** Comparison of Experimental Results of DIOR Datasets.

Method	PP-Loss	Image Size	FPN Layers	*P*	*R*	${\mathrm{mAP}}_{S}$	${\mathrm{mAP}}_{M}$	${\mathrm{mAP}}_{L}$	mAP	mAP50
YOLOv8s	No	640	3	0.883	0.756	0.270	0.697	0.840	0.626	0.820
YOLOv8s	Yes	640	3	0.902	0.764	0.305	0.708	0.842	0.640	0.826
YOLOv8s + 4H	No	640	4	0.888	0.752	0.264	0.692	0.838	0.621	0.816
YOLOv8s + 4H	Yes	640	4	0.900	0.765	0.273	0.694	0.835	0.640	0.829
YOLOv8 + AFPN	No	640	3	0.879	0.756	0.260	0.687	0.825	0.615	0.817
YOLOv8 + AFPN	Yes	640	3	0.892	0.758	0.276	0.690	0.824	0.620	0.821
ASF-YOLO	No	640	3	0.884	0.760	0.254	0.689	0.828	0.615	0.815
ASF-YOLO	Yes	640	3	0.899	0.767	0.286	0.692	0.839	0.627	0.825
YOLOv10s	No	640	3	0.895	0.738	0.283	0.694	0.844	0.628	0.818
YOLOv10s	Yes	640	3	0.904	0.754	0.307	0.705	0.853	0.641	0.823

**Table 3:** Comparison of the experimental effects between this algorithm and other algorithms that have improved the classification loss. The VisDrone dataset was used in the experiment.

Method	*P*	*R*	mAP	mAP50
YOLOv8s	0.520	0.392	0.241	0.404
YOLOv8s + SBL	0.534	0.394	0.256	0.414
Redet + ARUBA	/	/	0.203	0.328
YOLOv8s + PPLoss	0.532	0.399	0.258	0.417

**Table 4:** The impact of setting different parameter q on detection results using the VisDrone dataset.

Parameter σ	*P*	*R*	mAP	mAP50
No	0.520	0.392	0.241	0.404
50	0.512	0.389	0.238	0.401
100	0.515	0.396	0.235	0.401
200	0.532	0.399	0.258	0.417
300	0.522	0.400	0.239	0.407

**Table 5:** Comparison of the experimental results between using the same standard deviation and using different standard deviations.

Parameter σ	*P*	*R*	mAP	mAP50
No	0.520	0.392	0.241	0.404
$\sigma_{3}=\sigma_{4}=\sigma_{5}=200$	0.532	0.399	0.258	0.417
$\sigma_{3}=19$, $\sigma_{4}=24$, $\sigma_{5}=14$	0.474	0.361	0.214	0.368

## Data Availability

Data are contained within the article.

## Abbreviations

The following abbreviations are used in this manuscript:

PPF	Prediction Probability Function
PP-Loss	Prediction Probability Loss
